# The Neuroses of the Genitourinary System in the Male, with Sterility and Impotence

**Published:** 1902-10

**Authors:** 


The Neuroses of the Genitourinary System in the Male, with Sterility and Impotence. By Dr. R. Ultzmann, Professor of Genitourinary Diseases in the University of Vienna. Second edition. Revised, with notes and a supplementary article on Nervous Impotence, by the translator, Gardner W. Allen, M. D., Surgeon in the Genitourinary Department of the Boston Dispensary, Instructor in Genitourinary Surgery in Tuft's Medical College. Illustrated. 12mo, 198 pages. Philadelphia: F. B. Davis Company. 1902. [Price, $1.00 net.]

It speaks well for the genius of Ultzmann that although it is years since he wrote the monographs, which in the main make up this book, little of any value has been added to the subject, so great was his knowledge and so comprehensive his grasp of the details of diagnosis and treatment. True, there have been advances made in matters relating to sexual disorders, but they in no wise trespass on the ground Ultzmann originally covered; they merely add to the field of study. These monographs were translated by Dr. Allen and to them he has added notes which bring the whole subject-matter down to the present time, and make the book one of the most complete of its kind in the world. A broad statement maybe, for in these days of progress in all branches of medicine and surgery most excellent works are constantly being produced, yet the fact remains that as yet no one has improved on Ultzmann's work. None but genitourinary men could attempt it with any degree or hope of success, and Ultzmann left little but stubble in the field for them to harvest.

The introduction makes plain the connection between nervous affections of the urinary system and similar affections of the genital organs and shows clearly how they may be so interwoven one with the other that grave consequences may ensue. There is a brief and clear statement of facts concerning neuroses in general, confined, of course, to the limitations of the book, and then taken up in regular order comes a study of the urine in genitourinary neuroses. The sensory, motor and secretory neuroses of the urinary and sexual systems are considered separately. Sterility, impotence and nervous impotence are all gone into very thoroughly when the size of the volume is considered. Dr. Allen's additions to the text and his notes add to the value of the book as a work of assistance to the practitioner; and best of all the book is small, compact, precise and not overburdened with pictures or unnecessary "I's."

